# Relationship between leadership coaching and nurses’ resilience in hospital environments^*^

**DOI:** 10.1590/1980-220X-REEUSP-2022-0265en

**Published:** 2023-01-30

**Authors:** Heloísa de Góes Gigueira Menezes, Andrea Bernardes, Simone Coelho Amestoy, Isabel Cristina Kowal Olm Cunha, Maria Lúcia Alves Pereira Cardoso, Alexandre Pazetto Balsanelli

**Affiliations:** 1 Hospital do Servidor Público Estadual, São Paulo, SP, Brazil.; 2 Universidade de São Paulo, Escola de Enfermagem de Ribeirão Preto, Ribeirão Preto, SP, Brazil.; 3 Universidade Federal do Vale do São Francisco, Petrolina, PE, Brazil.; 4 Universidade Federal de São Paulo, Escola Paulista de Enfermagem, São Paulo, SP, Brazil.; 5 Hospital Alemão Oswaldo Cruz, São Paulo, SP, Brazil.

**Keywords:** Leadership, Resilience, Psychological, Nursing, Nursing Service, Hospital, Nursing Administration Research, Liderazgo, Resiliencia Psicológica, Enfermería, Servicio de Enfermería en Hospital, Investigación en Administración de Enfermería, Liderança, Resiliência Psicológica, Enfermagem, Serviço Hospitalar de Enfermagem, Pesquisa em Administração de Enfermagem

## Abstract

**Objective:**

To analyze the relationship between leadership coaching and nurses’ resilience in hospital environments.

**Method:**

This is a cross-sectional study, carried out with nurses, nursing assistants and technicians. Nurses answered the Nurse Self-Perception Questionnaire in Leadership Exercise Questionnaire (QUAPEEL) and the Connor-Davidson Scale for Brazil-25 (CD-RISC-25^BRASIL^). Nursing technicians/assistants answered the Nursing Technician and Assistant Perception in Leadership Exercise Questionnaire (QUEPTAEEL). Student’s t test and Pearson’s correlation were used (p ≤ 0.05).

**Results:**

There was a statistically significant difference between nurses’ self-perception and nursing technicians/assistants’ hetero-perception in coaching leadership exercise for the total score (p = 0.002) and in the “Give and receive feedback” (p < 0.001), “Delegate power and exert influence” (p < 0.001) and “Support the team so that the organizational results are reached” (p = 0.020) domains. There was a statistically significant correlation between all the Nurse Self-Perception Questionnaire in Leadership Exercise Questionnaire Coaching and Resilience domains.

**Conclusion:**

Resilience was positively related to nurses’ self-perception of coaching leadership.

## RESUMO

**Objetivo:**

Analisar a relação entre liderança *coaching* e resiliência dos enfermeiros no ambiente hospitalar.

**Método:**

Estudo transversal, realizado com enfermeiros, auxiliares e técnicos de enfermagem. Os enfermeiros responderam ao Questionário de Autopercepção do Enfermeiro no Exercício da Liderança (QUAPEEL) e à Escala de Connor-Davidson para o Brasil-25 (CD-RISC-25^BRASIL^). Os técnicos/auxiliares de enfermagem responderam ao Questionário de Percepção do Técnico e Auxiliar de Enfermagem no Exercício da Liderança (QUEPTAEEL). Os Testes t de Student e Correlação de Pearson foram utilizados (p ≤ 0,05).

**Resultados:**

Verificou-se diferença estatisticamente significante entre autopercepção dos enfermeiros e heteropercepção dos técnicos/auxiliares de enfermagem no exercício da liderança *coaching* para pontuação total (p = 0,002) e nos seus domínios “Dar e receber *feedback*” (p < 0,001), “Dar poder e exercer influência” (p < 0,001) e “Apoiar a equipe para o alcance dos resultados” (p = 0,020). Houve correlação estatisticamente significante entre todos os domínios do Questionário de Autopercepção do Enfermeiro no Exercício da Liderança *Coaching* e Resiliência.

**Conclusão:**

A resiliência se relacionou positivamente com a autopercepção de liderança *coaching* dos enfermeiros.

## RESUMEN

**Objetivo:**

Analizar la relación entre el coaching de liderazgo y la resiliencia de enfermeros en el ambiente hospitalario.

**Método:**

Estudio transversal, realizado con enfermeros, auxiliares y técnicos de enfermería. Los enfermeros respondieron el Cuestionario de Autopercepción de Enfermeros en el Ejercicio del Liderazgo (QUAPEEL) y la Escala de Connor-Davidson para Brasil-25 (CD-RISC-^BRASIL^). Los técnicos/auxiliares de enfermería respondieron el Cuestionario de Percepción del *Técnico y Auxiliar de* Enfermería en el Ejercicio del Liderazgo (QUEPTAEEL). Se utilizó la prueba t de Student y la Correlación de Pearson (p ≤ 0.05).

**Resultados:**

Hubo diferencia estadísticamente significativa entre la autopercepción de los enfermeros y la heteropercepción de los *técnicos/auxiliares de* enfermería en el ejercicio del liderazgo de coaching para la puntuación total (p = 0,002) y en los dominios “Dar y recibir feedback” (p < 0,001), “Dar poder y ejercer influencia” (p < 0,001) y “Apoyar al equipo para lograr resultados” (p = 0,020). Hubo correlación estadísticamente significativa entre todos los dominios del Cuestionario de Autopercepción del Enfermero en el Ejercicio del Liderazgo, Coaching y Resiliencia.

**Conclusión:**

La resiliencia se relacionó positivamente con la autopercepción de los enfermeros sobre el liderazgo en el entrenamiento.

## INTRODUCTION

The various changes experienced by current societies have reflected in workers’ daily lives, making them increasingly involved and charged by workplace demands and routines^(1)^. The work process in health is a complex and dynamic phenomenon, constantly influenced by changes in socioeconomic, political and technological scenarios^(2)^.

On a daily basis, nurses and other health team professionals are exposed to stressors, which can trigger physical and mental health problems, turnover of work teams, decreased professional performance and negative repercussions on direct nursing care to patients^(1–3)^.

In this context, nurses, as leaders of their teams, need to create working conditions that minimize these problems. Thus, coaching leadership and resilience, objects of investigation in this study, are considered relevant and essential competences for achieving organizational goals^(4)^, identified as constructively and positively influencing the results of nursing care^(5)^.

Coaching leadership is considered an innovative model in the health field. It is based on the mutual commitment between leaders (coach) and followers to achieve goals^(5–6)^. It is based on four domains: Communication; Give and receive feedback; Delegate power and exert influence; and Support the team so that the organizational results are reached organizational^(5–7)^.

The “Communication” domain is the process of understanding and sharing information in messages received and sent between leaders and followers, on an ongoing basis. Feedback is a tool used for developing work teams. The “Give and receive feedback” domain is a necessary skill for coaching leaders. Through it, the leader conveys information clearly to achieve professional goals. The “Delegate power and exert influence” domain consists of the power relationship that leaders exert over their followers in enabling them to achieve their goals and influencing their reactions. The “Supporting the team to achieve organizational results” domain is related to the alignment of organizational goals and individual expectations, taking into account their followers and the mutual trust between leader and follower^(6)^. Coaching leadership began to be adopted more frequently based on changes in the job market, which made people seek to improve their performance in order to contribute more effectively to institutional results^(8,9)^.

For coaching leadership exercise, it is important that leaders have a relationship of trust with their team, positively influencing their followers. Faced with this situation, this current and innovative model of leadership has proven to be an interesting alternative in the process of developing skills that are interrelated with leadership, such as the ability to relate, to work as a team, to communicate, to be resilient, among others^(10)^.

Resilience, which is also the object of investigation in this research, is defined as an individual’s ability to recover from adversity and adapt positively in situations of tension and stress. It is a concept widely used to explain the different effects that the same level of stress has on different individuals^(7)^. This mechanism of recovery and adaptation, in the face of adverse situations, has been recognized as an important aspect for mental health promotion and protection, reducing the intensity of stress and decreasing negative emotional signals, such as anxiety, depression and anger. It is a dynamic process in which the influences of the environment and individuals reciprocally interact, allowing it to adapt, despite the stressors^(1)^.

Faced with the adversities experienced by nurses while working in hospitals, it appears that resilience can be a valuable and fundamental resource in daily work, and can contribute to the exercise of leadership with greater efficiency^(3)^, while it can be considered an essential tool for nurses, even more so in the current context, in which changes and stressful situations have become constant, and knowing how to manage them is essential, due to their repercussions on professionals’ professional and personal lives.

In nursing, what is observed is that there is a shortage of studies, mainly that address nurses’ resilience and leadership, mainly in the national scenario, where the interface between resilience and leadership has still been approached in an incipient way, which corroborates the relevance of this study.

It is observed that resilience and coaching leadership are important characteristics for these professionals’ daily work, especially in hospital environments, where stressors and occupational stress can be more intense, with repercussions on nurses’ performance and quality of care provided to patients. Furthermore, in the area of nursing, there is a shortage of studies that address the relationship between resilience and leadership among nurses, which justifies the development of this study.

From the above, the following questions arose: what is the relationship between coaching leadership and nurses’ resilience in hospital environments? Is there a difference in these professionals regarding self and hetero-perception of coaching leadership exercise assessment? What is the nurse-leaders’ resilience score? Thus, the study aimed to analyze the relationship between coaching leadership and nurses’ resilience in hospital environments.

## METHOD

### Study Design

This is a cross-sectional, correlational and observational study, guided by the STrengthening the Reporting of OBservational studies in Epidemiology (STROBE) recommendations^(11)^.

### Site

Large hospital institution, characterized as an autonomous non-profit entity, with legal personality and own assets that serves public servants of the state of São Paulo, their dependents and associates, located in the municipality of São Paulo, SP, Brazil.

### Population

The population consisted of nurses and nursing technicians/assistants who worked in Hospital Admission (COVID and Non-COVID Infirmary Units), Hemodialysis, Blood Bank, Adult Emergency Room, Child Emergency Room and Chemotherapy Units.

### Selection Criteria

Participants met the following inclusion criteria: working at the institution for at least six months; being directly subordinated to nursing management; and being present during the data collection period. Initially, nurses were approached, and, after collecting data from these nurses, nursing assistants and technicians who made up their team were approached. If they agreed to participate in the study, they were randomly selected by the researcher and carried out an assessment of nurses’ leadership. Data collection was carried out from January to April 2021.

Exclusion criteria included: nursing professionals away from work during the data collection period due to vacation, leave (medical, disgust, gala, for example) or on-duty staff hired only to cover shifts.

### Sample

For sample size planning, the Analysis of Variance (ANOVA) technique was used, with 1 factor and r = 4 levels. It was then suggested to use a sample of 144 nurses and 144 nursing assistants/technicians, totaling 288 professionals. A total of 230 professionals participated in the study, 115 nurses and 115 nursing assistants/technicians, as they agreed to participate and met the inclusion criteria.

### Data Collection

Nurses answered the Nurse Self-Perception Questionnaire in Leadership Exercise Questionnaire (*Questionário de Autopercepção do Enfermeiro no Exercício da Liderança*, QUAPEEL) and the Connor-Davidson Scale for Brazil-25 (CD-RISC-25^BRASIL^). Nursing technicians/assistants answered the Nursing Technician and Assistant Perception in Leadership Exercise Questionnaire (*Questionário de Percepção do Técnico e Auxiliar de Enfermagem no Exercício da Liderança*, QUEPTAEEL).

QUAPEEL and QUEPTAEEL have structured questions, divided into three parts. The first addresses sociodemographic and professional data; the second part deals with issues related to knowledge of leadership by nurses in coaching leadership practice (open-ended and alternative questions about how participants conceptualize leadership and self-perception of coaching leadership exercise) and leadership exercise assessment by nurses by nursing assistants and technicians; and the third part addresses the four domains of coaching leadership (Communication; Give and receive feedback; Delegate power and exert influence; and Support the team so that the organizational results are reached), arranged in 20 items in total, 4 items for each domain. The items were measured using a 5-point Likert-type scale, with a score from 0 to 100. Each question includes response options from “never” to “always”, with “never” = 0 points and “always” = 5 points, and the higher the score, the greater the coaching leadership competence that professional has^(6)^. The two questionnaires were constructed and validated in Brazil. The Cronbach’s alpha value of the nurses’ self-perception instrument was 0.911, and the perception of nursing technicians’ and assistants’ perception, 0.932^(6)^.

The CD-RISC-25^BRASIL^ is a widely used version of the CD-RISC questionnaire for measuring resilience. It is an adaptation and validity for Brazilian culture with an alpha coefficient of 0.93 and intraclass correlation of 0.84^(12)^. It is a questionnaire with 25 questions, covering five domains, each with 5 items: Trust in one’s instincts and tolerance of negative affect; Positive acceptance of change; Control; Personal competence; and Spirituality. The answers are given in a Likert-type scale format, with 5 options to choose from: 0 = Not at all true; 1 = Rarely true; 2 = Sometimes true; 3 = Often true; and 4 = Almost always true. The score is based on the sum total of all items, whose scores range from 0 to 100, with higher scores demonstrating greater resilience scores^(12)^.

### Data Analysis and Treatment 

After collection, the data were organized and entered into Microsoft Excel^®^ spreadsheets by the researcher herself. Then, the database was checked, revised and validated by another professional.

Data were analyzed using descriptive statistics using IBM SPSS Statistics 26.0 for Microsoft Windows^®^ and R, version 4.0.3. Means, standard deviations, medians, minimum and maximum scores of quantitative variables were used.

In order to compare the scores obtained by nurses in their self-assessment in the exercise of leadership (QUAPEEL) and the hetero-assessment carried out by nursing assistants or technicians (QUEPTAEEL), Student’s t test was performed for independent samples, and the effect size was calculated using the d coefficient^(13)^, considering: small: between |0,200| and |0.499|; medium: between |0.500| and |0.799|; and large: above |0.800|^(13)^.

To carry out the investigation of the presence of correlation between coaching leadership (QUAPEEL and QUEPTAEEL) and resilience scores (CD-RISC-25^BRASIL^), Pearson’s correlation test was performed for the total sample. The statistical significance value adopted was equal to 5% (p ≤ 0.05)^(13)^.

### Ethical Aspects

The study was approved by the Research Ethic Committees of the *Universidade Federal de São Paulo*, under Opinion 4,355,763, of October 22, 2020, and of the co-participating institution, under Opinion 4,478,548, of December 21, 2020, with a view to meeting the determinations of Resolution 466/2012 of the Brazilian National Health Council. All participants signed the Informed Consent Form (ICF).

## RESULTS

A total of 115 nurses aged between 35 and 55 years participated in the survey. The mean age was 42 years (SD = 8.6); 92 (80%) were female, with an average training time of 11 years (SD = 6.9) and an average time working at the institution of 8 years (SD = 6.8). Table 1 presents the occupational sociodemographic characteristics of professionals who participated in the study.

**Table 1:** Occupational sociodemographic characteristics of nurses, nursing assistants and technicians – São Paulo, SP, Brazil, 2022.

**Category**	**N**	**%**
Nurses	115	50%
Nursing technicians/assistants	115	50%
**Age**		
**Nurses**		
25–34	19	16.5%
35–44	56	48.6%
45–54	27	23.4%
65	1	1%
**Nursing assistants/technicians**		
21–34	14	12.2%
35–44	42	36.5%
45–54	42	36.6%
55–64	17	14.8%
**Sex**		
**Nurses**		
Female	92	80%
Male	23	20%
**Nursing assistants/technicians**		
Female	94	82%
Male	21	18%
**Job tenure (years)**		
**Nurses**		
0.9–20	103	89.6%
21–42	12	10.4%
**Nursing assistants/technicians**		
1–15	85	74%
16–31	30	26%
**Working time**		
**Nurses**		
Morning	23	20%
Afternoon	3	3%
Intermediate	37	32%
Night	52	45%
**Nursing assistants/technicians**		
Morning	11	10%
Afternoon	0	0%
Intermediate	51	44%
Night	53	46%

Source: developed by the researcher.

As for the sector where nurses worked, 59 (51%) performed their activities in Non-COVID Inpatient, 22 (19%) COVID Inpatient, 22 (19%) Adult, Child and Hemodialysis Emergency, and 12 (10%) in Blood Bank and Chemotherapy Units.

With regard to the training of nurses, 86 (75%) had at least one *lato sensu* graduate course. None of the participants had a *stricto sensu* master’s or doctoral degree. Only 13 nurses (11.3%) had a specialization course in nursing management; 3 nurses (3%) were certified in public management, 2 (2%) in health management and 8 (7%) in hospital administration. The other nurses were certified in other areas of knowledge, such as intensive care, pediatrics and neonatology, nephrology, cardiology, public health and other areas.

Regarding nurses’ self-perception in exercise of coaching leadership, 112 (97%) acknowledged being leaders of their teams. When nurses were asked about the concept of leadership, 77 (67%) responded that it is the process of influencing people’s behavior to achieve goals in certain situations; 26 (23%) conceptualized leadership as the process of transforming an individual’s or an organization’s behavior; and 12 (10%) defined leadership in their own words. Regarding the leadership style exercised by nurses, it was found that the majority identified with people- and task-oriented leadership, depending on the situation in which they are involved.

Considering the main skills related to the communication process between leaders and followers, nurses selected the best answers thinking about skills they thought were most important. Of the alternatives offered in the instrument used during the research (1 – Communication skills; 2 – Ability to give and receive feedback; 3 – Ability to delegate power and exert influence; and 4 – All the skills mentioned above), the alternative most chosen by nurses was the option that all skills are necessary for leaders (38.2%; n = 44). Some nurses (32.1%; n = 37) also chose two other joint responses, namely: Communication skills; and Ability to give and receive feedback. Last choice was the answer Ability to gain power and exert influence, selected by 34 (29.5%) professionals.

Regarding the total score of the scores of coaching leadership domains, the minimum value was 49 and the maximum, 100 points, with an average of 84.41 (SD = 9.44), which demonstrates that there is a diversified degree of leadership coaching development for nurses in the institution.

Part of the study consisted of assessing nurses’ resilience score in this institution. To this end, the CD-RISC-25^BRASIL^ was used. Regarding the sum of participants’ total resilience scores, the minimum value of the sum of the scores was 45 and the maximum value was 97, with an average of 77 (SD = 10.51).

During participation in the study, nursing technicians and assistants were asked about their knowledge of the concept of leadership and whether these professionals considered their immediate superiors as leaders. Most nursing technicians and assistants (88%; n = 101) considered nurses leaders, and only 14 (12%) of these professionals reported not considering their immediate superiors a reference in leadership. Regarding the total scores related to assessment of nurses’ coaching leadership, carried out by nursing technicians and assistants, it was found that the minimum value was 12 and the maximum value was 100 points, with an average of 77.61 (SD = 21.74), which demonstrates disagreement with the self-perception of the assessment carried out by nurses. Table 2 presents the measures of central tendency and dispersion of the scores obtained by QUAPEEL (self-assessment) and QUEPTAEEL (inter-assessment) nurses, as well as the comparison of scores in both conditions using Student’s t test, for independent samples, and effect size.

**Table 2:** Comparison of Nurse Self-Perception Questionnaire in Leadership Exercise Questionnaire and Nursing Technician and Assistant Perception in Leadership Exercise Questionnaire scores – São Paulo, SP, Brazil, 2022.

**Score**	**Assessment**	**n**	**Mean**	**SD**	**Median**	**Minimum**	**Maximum**	**p**	**E.S.**
Total	Self	115	84.41 [82.70. 86.05]	9.44	86.00 [85.00. 86.00]	49.00	100.00	0.002*	0.406
	Hetero	115	77.61 [73.52. 81.38]	21.74	85.00 [79.00. 91.00]	12.00	100.00		
Communication	Self	115	21.70 [21.24. 22.10]	2.38	22.00 [22.00. 22.00]	11.00	25.00	0.714	0.048
	Hetero	115	21.52 [20.69. 22.29]	4.49	24.00 [24.00. 24.00]	6.00	25.00		
Give and receive feedback	Self	115	21.87 [21.43. 22.30]	2.38	22.00 [22.00. 22.00]	12.00	25.00	< 0.001*	0.510
	Hetero	115	19.80 [18.83. 20.71]	5.23	22.00 [20. 22.00]	6.00	25.00		
Delegate power and exert influence	Self	115	21.17 [20.60. 21.67]	2.97	22.00 [22.00. 22.00]	7.00	25.00	< 0.001*	0.528
	Hetero	115	18.32 [17.09. 19.50]	7.01	21.00 [20.00. 22.00]	0.00	25.00		
Support the team	Self	115	19.68 [18.98. 20.34]	3.85	20.00 [20.00. 21.00]	7.00	25.00	0.020*	0.310
	Hetero	115	17.97 [16.69. 19.17]	6.79	20.00 [19.00. 22.00]	0.00	25.00		

E.S. – effect size; SD – standard deviation. Source: elaborated by the researcher.

The “Give and receive feedback” and “Power and exert influence” domains had effect sizes classified as medium according to the coefficient d^(13)^. The others were considered small.

Table 3 presents the correlation analysis between the scores obtained by nurses in QUAPEEL and QUEPTAEEL and CD-RISC-25^BRASIL^ scores. For this analysis, the correlation coefficient and p-value were calculated, through Pearson’s correlation test for the total sample, since the sample presented “n” large enough for the direct use of parametric tests, due to the Central Limit Theorem.

**Table 3.** Correlation analysis between the scores obtained by nurses in the Nurse Self-Perception Questionnaire in Leadership Exercise Questionnaire and in the Nursing Technician and Assistant Perception in Leadership Exercise Questionnaire and on the Connor-Davidson Scale score for Brazil-25 – São Paulo, SP, Brazil, 2022.

**Instrument**		**QUAPEEL**				
		**Total**	**1**	**2**	**3**	**4**
CD-RISC-25^BRASIL^	Coef.	**0.293** **[0.099. 0.470]**	**0.265** **[0.043. 0.451]**	**0.262** **[0.083. 0.431]**	**0.277** **[0.104. 0.444]**	0.178 [–0.008. 0.360]
	P	0.001*	0.004*	0.005*	0.003*	0.057
		**QUEPTAEEL**				
		**Total**	**1**	**2**	**3**	**4**
CD-RISC-25^BRASIL^	Coef.	0.009 [–0.161. 0.175]	–0.004 [–0.175. 0.173]	0.007 [–0.171. 0.189]	–0.038 [–0.197. 0.122]	0.065 [–0.116. 0.233]
	P	0.923	0.966	0.938	0.689	0.490

Pearson’s correlation test; coef. – coefficient; QUAPEEL – Nurse Self-Perception Questionnaire in Leadership Exercise Questionnaire; QUEPTAEEL – Nursing Technician and gAssistant Perception Questionnaire in the Exercise of Leadership; CD-RISC-25^BRASIL^ – Connor-Davidson Scale for Brazil-25; *Statistically significant value at the 5% level (p ≤ 0.05); 1 – Communication; 2 – Give and receive feedback; 3 – Delegate power and exert influence; 4 – Support the team so that the organizational results are reached. Source: elaborated by the researcher.

## DISCUSSION

Most nurses recognized themselves as leaders of their teams, and believe that leading is exerting influence on people’s behavior to achieve goals in certain situations and transform the behavior of an individual or an organization.

It is through leadership that nurses promote the quality of nursing care, as they motivate the team to accept new practices^(14,15)^. This conception of leadership moves towards a contingency view, in which nurses analyze the various dimensions that permeate a situation, to guide their leadership style and make more assertive and coherent decisions with the scenario and the problem experienced.

In this context, the importance of leadership is emphasized as an indispensable and necessary competence for nurses’ practice, as leaders inspire and encourage their teams to accept innovation and transformation actions. It encourages the bond of trust between leader and follower, contributing to the quality of nursing care provided to patients, which reflects on job satisfaction. Moreover, it is necessary for leaders to adapt their behavior to the teams’ context and reality, as leadership influences the work environment^(16,17,18,19)^.

In a study on situational leadership styles adopted by nurses in a hospital area, it is mentioned that the influence of leadership styles is used to qualify care, and when it has high quality, it is full of trust, good communication, respect and reciprocity^(17)^. When there are coaching leadership characteristics in nurses, there is job satisfaction, demonstrated by mutual trust, continuous interaction between leaders and followers, and the search for professional and personal development^(16,17)^, which reinforces the importance of investing in and encouraging the implementation of programs to develop these skills in nurses in hospital environments as a strategic objective viscerally in organizational culture.

The results indicated that most nurses had, at least, a *lato sensu* graduate course, however, a scarce number related to the management area. Furthermore, it is also noteworthy that none of the participants had a *stricto sensu* master’s or doctoral degree. This finding alerts to the need for greater investment by the institution to enhance professional development, given that research carried out in the academy serves as subsidies for practices developed in hospital environments, impacting service quality. With regard to nurses’ self-perception (QUAPEEL) and assessment of nursing assistants’ and technicians’ perception in the exercise of nursing leadership coaching (QUEPTAEEL), in this study, divergences between the assessments (self and hetero-perceptions) were evidenced.

Nurses’ scores were higher when compared to the assessment that followers made about these professionals’ coaching leadership exercise. Such differences in assessment perception were also found in two other studies, in which coaching leadership was shown with higher scores in nurses’ self-perception (leaders) than in followers’ self-perception (nursing assistants/technicians)^(20–21)^. One of these studies mentions that this fact occurs because nurses are more aware of the exercise of their influence on their followers. It must be considered that there is evidence that the self-assessment performed by professionals is always more positive, which can be considered a bias^(21)^.

The domains that showed a statistically significant difference between the self- and hetero-assessment conditions in the study were “Give and receive feedback”, “Delegate power and exert influence” and “Support the team so that the organizational results are reached” and the total score. There was concordance of assessments in the “Communication” domains.

A study carried out in two hospitals in São Paulo^(15)^, aiming at identifying and comparing nurses’ self-perception and nursing assistants’ and technicians’ perception regarding coaching leadership practices, also showed divergence in the perception between these two categories. Similar to the findings of this study, the results of the study carried out in these hospitals^(21)^ showed that there was divergence in “Give and receive feedback”, “Delegate power and exert influence” and “Support the team so that the organizational results are reached” as well as the total coaching leadership score. There was agreement in the “Communication” domain between nursing teams in the two hospital units^(15)^.

In another study, carried out in two university hospitals and two private hospitals^(6)^, the findings also showed that there was a statistically significant difference between leaders’ and followers’ perception. The same also occurred in a study carried out outside hospital units, in the Mobile Emergency Care Service^(22)^, and in a study carried out with professionals in Primary Health Care^(23)^.

The “Communication” domain was recognized as a present characteristic and recognized by both categories (leaders and followers). In the cited studies^(6,20–22)^, there is an agreement of self and hetero-perception of this characteristic in coaching leadership exercise.

Communication is recognized as essential in nurses’ daily lives, as it can influence workers’ behavior and performance in achieving goals. It is also a working tool, since nurses often perform articulation functions between other teams and professionals for human care^(23–24)^. The study points to the importance of communication as a basic element of coaching leadership as well as the need to reduce noise and encourage employees’ active participation in decision-making processes.

Regarding the resilience score of nurses participating in the study, it was found that most professionals had a score equal to or greater than 70 points, which can be considered resilient. Corroborating this finding, what is observed in practice are the presence and performance of dedicated professionals who, even going through difficulties that are particular to nurses’ work, they know how to deal with conflicts and manage the demands that arise in an optimistically and altruistically. Regarding the variables nurses’ resilience and self-perception regarding coaching leadership exercise (QUAPEEL), in this study, it was found that there is a statistically significant and directly proportional correlation, and the increase in one variable was associated with the increase in another, i.e., the higher the QUAPEEL score, the higher the leader coach resilience score.

Thus, investing in coaching leadership can be an interesting strategy for institutions, as coaching leaders prove to be resilient. In a context of health work, especially in the pandemic and post-pandemic reality imposed by COVID-19, this seems to be a strategic decision for managers, because, in this context, coach leaders naturally act as educators, work with the development of their teams, investigate and encourage everyone’s evolution, managing to deal with the adversities and stressors of everyday life^(22,25)^. International studies have shown extremely positive changes after adopting leadership coaching programs. In Australia, the program was associated with significant improvements in goal achievement, and employees became more solution-focused, increased self-awareness, resilience and tolerance. Study participants reported being able to use the lessons learned in coaching, having a better balance between work and personal life^(26)^.

In a study carried out in London, with nursing managers, after adopting the coaching methodology, nurse managers became more resilient and confident, as well as acquired better mechanisms for coping with conflict situations. This allowed better management and team cohesion perception, leading to better quality of patient care^(27)^.

Likewise, in Spain, in a study carried out with executives from an automobile sector, similarly to what can happen in hospitals, a similar result was observed, whereas, after specialized intervention and leadership coaching program training, the results were positive and helped leaders to develop coaching skills, improve professionals’ well-being and performance in organizations^(28)^.

In this regard, the relationship of the coaching leadership model associated with resilience was verified as an advance. Therefore, this model can be very interesting for people management in institutions, for offering greater robustness and scientificity in the development of leaders in nursing, with positive repercussions on qualification of care, professional satisfaction and organizational climate.

As a limitation of this study, it is noteworthy that the analysis of the relationship between coaching leadership and nurses’ resilience in hospital environments occurs in a single context, characterizing a specific population’s perception. Thus, further studies on the subject are suggested to expand this knowledge and strengthen studies of management and leadership in nursing.

## CONCLUSION

From this study, it can be concluded that there is a difference between the self-perception of leaders in relation to the exercise of coaching leadership assessed by followers. Such differences in assessments were statistically significant in the total score of coaching leadership, in “Give and receive feedback”, “Delegate power and exert influence” and “Support the team to achieve results”.

With regard to resilience, according to the scores obtained by the nurses participating in the study, they can be considered resilient. Furthermore, resilience was shown to be present in leaders, with an increase in one variable (QUAPEL score) being associated with an increase in another variable (CD-RISC-25^BRASIL^), observing a statistically significant correlation. Such correlations occurred in the total QUAPEEL and CD-RISC-25^BRASIL^ scores, with the domains: “Communication” and CD-RISC-25^BRASIL^; “Give and receive feedback” and CD-RISC-25BRASIL; and “Delegate power and exert influence” and CD-RISC-25^BRASIL^.

In this way, leadership coaching, as it is a model in which leaders seek to achieve results by encouraging the development of followers, is a valuable strategy for people management to be adopted by health institutions. Moreover, it can increase nurses’ resilience to overcome challenging situations experienced in hospital environments.
